# Impact of COVID-19 infection on life expectancy, premature mortality, and DALY in Maharashtra, India

**DOI:** 10.1186/s12879-021-06026-6

**Published:** 2021-04-12

**Authors:** Guru Vasishtha, Sanjay K. Mohanty, Udaya S. Mishra, Manisha Dubey, Umakanta Sahoo

**Affiliations:** 1 International Institute for Population Sciences, Govandi Station Road, Deonar, Mumbai, Maharashtra 400088 India; 2 Department of Fertility Studies, International Institute for Population Sciences, Mumbai, India; 3 Centre for Development Studies, Prashant Nagar, Medical College P.O, Ullor Thiruvananthapuram, Kerala India; 4 Centre for Chronic Disease Control, New Delhi, India

**Keywords:** COVID-19, Premature mortality, Life expectancy, YPLL, DALY, Maharashtra

## Abstract

**Background:**

The COVID-19 infections and deaths have largely been uneven within and between countries. With 17% of the world’s population, India has so far had 13% of global COVID-19 infections and 8.5% of deaths. Maharashtra accounting for 9% of India’s population, is the worst affected state, with 19% of infections and 33% of total deaths in the country until 23rd December 2020. Though a number of studies have examined the vulnerability to and spread of COVID-19 and its effect on mortality, no attempt has been made to understand its impact on mortality in the states of India.

**Method:**

Using data from multiple sources and under the assumption that COVID-19 deaths are additional deaths in the population, this paper examined the impact of the disease on premature mortality, loss of life expectancy, years of potential life lost (YPLL), and disability-adjusted life years (DALY) in Maharashtra. Descriptive statistics, a set of abridged life tables, YPLL, and DALY were used in the analysis. Estimates of mortality indices were compared pre- and during COVID-19.

**Result:**

COVID-19 attributable deaths account for 5.3% of total deaths in the state and have reduced the life expectancy at birth by 0.8 years, from 73.2 years in the pre-COVID-19 period to 72.4 years by the end of 2020. If COVID-19 attributable deaths increase to 10% of total deaths, life expectancy at birth will likely reduce by 1.4 years. The probability of death in 20–64 years of age (the prime working-age group) has increased from 0.15 to 0.16 due to COVID-19. There has been 1.06 million additional loss of years (YPLL) in the state, and DALY due to COVID-19 has been estimated to be 6 per thousand.

**Conclusion:**

COVID-19 has increased premature mortality, YPLL, and DALY and has reduced life expectancy at every age in Maharashtra.

**Supplementary Information:**

The online version contains supplementary material available at 10.1186/s12879-021-06026-6.

## Introduction

In a short span of 1 year, COVID-19 has emerged as the largest-ever health crisis of the twenty-first century. With over 78 million infections and 1.7 million deaths attributable to it until 23rd December 2020, COVID-19 attributable deaths account for 2.9% of additional deaths worldwide [1, 2]. The global spread of COVID-19 infection and attributable mortality has been highly uneven among and within countries. With 18.6 million infections and 3,30,824 COVID-19 attributable deaths, the USA accounts for 23.8% of global infections and 19.2% of global deaths [1]. India, with over 10 million infected cases and 1,46,476 COVID-19 deaths, is the second-largest country with respect to the size of infection and is ranked third with respect to COVID-19 attributable deaths [1]. The actual number of infections in many countries, including India, remains underestimated due to the asymptomatic nature of the infection and inadequate testing and surveillance system.

As the COVID-19 infection continues to spread, an increasing number of studies have become available on the extent of infection, the associated risk factors, and the crude fatality ratio (CFR) with and without time lag, projecting deaths and estimating the loss of life expectancy, premature mortality, and YPLL across countries [3–9]. Findings suggest that the infection rate across populations is largely underestimated, while the CFR shows large variations across countries, geographies, and demographic characteristics. The demographic structure, availability of health care resources, and multimorbid conditions explain COVID-19 attributable deaths to a larger extent [3, 10–14]. In China, fever, dyspnea, and chest pain/discomfort have been the more common symptom among the deceased patients, while fever has the most common symptom among the surviving patients [3, 10]. Older adults, people with comorbidities, and men are more susceptible to COVID-19 fatality [11, 15].

Evidence suggests that people with the COVID-19 infection are more prone to many life-threatening morbidities and fatalities [11, 16]. A study conducted in Italy found that fatigue, dyspnea, joint pain, and chest pain were persistent among the recovered patients [16]. Studies have projected premature mortality and reduction in life expectancy due to the infection across countries [4, 8, 17, 18]. After a certain threshold level of COVID-19 prevalence, life expectancy starts decreasing. In North America, Europe, Latin America, and the Caribbean, life expectancy at birth has been estimated to have reduced by 1 year at 10% prevalence of infection [4]. The COVID-19 attributable mortality has the potential to reduce life expectancy in India, weekly and annual life expectancy at birth in Spain, and seasonal life expectancy in Italy [8, 17, 18]. Besides mortality, many studies are available on the vulnerability to the COVID-19 infection, and mental distress, and loss of livelihood due to the preventive measures for containing the virus [19, 20].

The spread of COVID-19 has been largely uneven across the states of India. With 123 million population (9% of India’s population), Maharashtra is the second most populous and urbanized state in the country. It is one of the more developed states and ranks high on the human development index [21]. However, Maharashtra is the worst affected state with respect to COVID-19 infections and mortality. Until 23rd December 2020, it had 1.9 million cases and 48,876 deaths due to COVID-19, accounting for 19% of total infections and 34% of all COVID-19 attributable deaths in the country [22]. The case-fatality ratio in the state is higher than the national average. It has been observed that the rapid community transmission of the virus in a short time has resulted in a higher incidence of the disease and deaths resulting from it and, consequently, has affected the life expectancy [12]. Many states, including Maharashtra, are now experiencing the second and the third waves of the COVID-19 pandemic. With the global literature hinting at the implications of COVID-19 for longevity, it becomes imperative to make a regional assessment of the same owing to the disproportionately high load of infections and deaths due to the pandemic in the region. This assessment involves premature mortality, with its consequential bearing on life expectancy, person-years of life lost, and disability-adjusted life years (DALY). With the age-specific load of the infection and fatalities, person-years of life lost offers an understanding into the skewed share of life lost during the productive years, which has implications not only for a macro assessment, but also for household-level micro assessment. Years of potential life lost (YPLL) is a summary measure of premature mortality that reflects the sum of years lost from a predefined age, such as standard life expectancy. A higher YPLL is indicative of premature mortality and contributes to the compression of life expectancy. DALY measures the disease burden of the population and consists of YPLL and Years Lived with Disability (YLD). DALY serves to understand the implications of differential severity of the disease for individuals conditioned by their age, sex, and any pre-disposed condition. In the context of the COVID-19 pandemic, estimating YPLL and DALY is appropriate as over two-thirds of deaths are under 70 years of age ‘a standard age for estimating YPLL’ [2]. Patients affected by COVID-19 have long-term health complications and are more likely to be morbid than non-COVID-19 patients [23]. In ultimate terms, the loss of life expectancy in a regional setting reflects the severity of the pandemic with sustained and periodic soaring of infection in the state. In this context, this paper examines the effect of COVID-19 on premature mortality, life expectancy, YPLL, and DALY in one of the worst affected states of India, Maharashtra.

### Data and methods

Data for this paper was drawn from multiple sources. These include the Report of the Expert Committee on Population Projections, Sample Registration System (SRS) Statistical Report 2018, and other published sources. The population size and distribution for Maharashtra for the year 2020 were taken from the Report of the Expert Committee on Population Projections [24]. The age-specific death rates for the state for the year 2018 (latest available data) were taken from the SRS Statistical Report and labelled as death rate without the COVID-19 infection [25]. The COVID-19 confirmed cases and deaths by age group were taken from the Times of India reports, dated 7th December 2020 and 21st December 2020 [26, 27]. The total number of confirmed cases and deaths until 20th December 2020 for Maharashtra and India were taken from covid19india.org [22]. We redistributed the total deaths until 20th December 2020, as per the distribution of deaths for which age data was available (7th December 2020). Age-specific case fatality ratio (ASCFR) was computed from the given data.

## Methods

Descriptive statistics, abridged life tables, and estimates of YPLL and DALY were used in the analysis. It was assumed that COVID-19 attributable deaths are unprecedented additional deaths that could have been averted in the absence of COVID-19. A set of abridged life tables were generated to estimate life expectancy at various ages, premature mortality, and YPLL and DALY were used in the analysis. The probability of death by age 70 and between age 20 and age 64 was computed and termed as premature mortality. Estimates were classified under the five scenarios. Scenario 1 provides estimates of deaths without COVID-19. Scenario 2 considers that COVID-19 deaths accounted for 5.3% of total deaths until 20th December 2020. Scenarios 3, 4, and 5 project the estimates assuming that COVID-19 attributable deaths would increases to 6, 8 and 10% of total deaths respectively. Expected deaths due to COVID-19 were distributed in accordance with the age distribution of COVID-19 as of date. Estimates of YPLL and DALY were made as follows.

### Years of potential life lost (YPLL)

YPLL is a summary measure of premature mortality that estimates the average years a person would have lived had he or she not died prematurely [28]. YPLL is estimated as:

$$ YPLL=\sum \limits_{i=0}^{\infty }{d}_i\ast {L}_i $$

Where *L*_*i*_ represents the life expectancy at age *i* and *d*_*i*_ represents the number of deaths at age *i*. It is a self-weighted estimate, which gives a higher weight to the deaths occurring at younger ages and a lower weight to the deaths at higher ages [28, 29]. The deaths at each age are weighted by age-specific life expectancy.

### Disability adjusted life years (DALY)

DALY is a summary measure of health of a population, combining mortality and non-fatal health outcomes. DALY is commonly used to measure the difference between a current situation of health and an ideal situation, where everyone lives up to the age of the standard life expectancy and in a perfect health [30]. It is estimated by summing the potential life lost due to premature mortality and productive years of life lost due to disability/disease [30]. It is calculated as:

$$ DALY= YLL+ YLD $$

Where *YLL* denotes the years of life lost due to premature mortality, and *YLD* denotes the years lived with disability.

YLL and YLD were calculated considering the discounting rate of 3%. Discounting rate shows the social preference of a healthy year now, rather than in the future. The value of a year of life is generally decreased annually by a fixed percentage. The World Bank Disease Control Priorities study and the Global Burden of Disease (GBD) project both used a 3% discount rate, and the US Panel on Cost-Effectiveness in Health and Medicine recently recommended that the economic analyses of health also use a 3% real discount rate to adjust both costs and health outcomes [31, 32].

YLL is estimated as:

$$ YLL=\frac{N}{r}\left(1-{e}^{- rL}\right) $$

Where *N* is the number of deaths, *L* is the life expectancy at the age of death, and *r* is the discount rate.

$$ YLD=\frac{\left(I\ast DW\ast L\ast \left(1-{e}^{- rL}\right)\right)}{r} $$

Where *I* is the number of incidence/prevalence cases, *DW* is a disability weight (a weight factor that reflects the severity of the disease on a scale from 0 (equivalent to perfect health) to 1 (equivalent to being dead)), and *L* is the duration of the disability.

As COVID-19 is a novel disease, its disability weight is not available. Since it is a severely infectious disease having an acute period and is associated with the lower respiratory tract infection [33]. Hence, we have used the disability weight of 0.133, as a proxy of COVID-19 (available elsewhere [34]. The duration of disability of 60 days was used because the patients of COVID-19 have been hospitalized for an average of 30 days, and after discharge, are quarantined for 14–28 days approximately [35, 36].

## Result

Table 1 provides the key indicators of the COVID-19 infection and the associated mortality for Maharashtra and India. Since the onset of the pandemic, COVID-19 has infected more than 1.8 million people, of whom 48,746 had died in Maharashtra until 20th December 2020. COVID-19 attributable deaths amount to 5.3% of the total deaths. These additional deaths could have been prevented in the absence of COVID-19. The case-fatality ratio in the state is 2.57, higher than the national average of 1.5. The rate of infection in Maharashtra (15 infected per 1000 people) is more than double compared to the national average (7 infected per 1000 people). In 2018 (the pre-COVID-19 period), the life expectancy at birth was 73.2 years in the state compared to 69.7 years in India as a whole.

**Table 1:** Summary indicators of population and COVID-19 indicators in Maharashtra and India 2020

Summary Indicators	Maharashtra	India
Total Population in 000 s (2020)	123,961	1,355,417
Estimated number of deaths without COVID-19	872,081	12,548,843
Number of deaths with COVID19 (until 20th December 2020)	48,746	145,845
Total deaths including deaths due to COVID-19	920,827	12,694,688
COVID-19 deaths as a share of total deaths until 20th December 2020	5.3	1.15
Case-fatality ratio	2.57	1.45
Total number of COVID-19 infection until 20th December 2020	1,896,518	10,056,281
Infection rate (per thousand population) until 20th December 2020	15.3	7.4
Life expectancy pre-COVID-19	73.2	69.7
Reduction in life expectancy with COVID-19 deaths accounting to 5.3%	0.8	NA

**Fig. 1:**
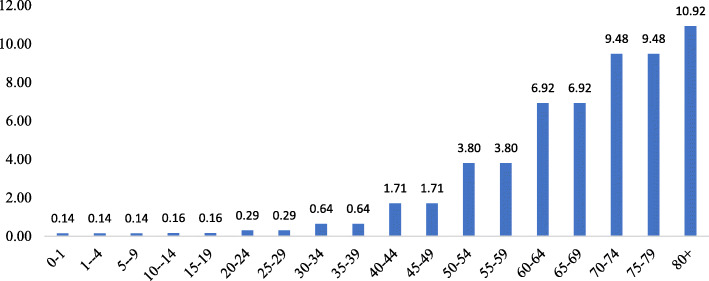
Age-specific case fatality ratio in Maharashtra, India 2020

Appendix 1 presents the number of confirmed cases and COVID-19 attributable deaths by age group in Maharashtra until 20th December 2020. Figure 1 estimates the age-specific case fatality ratio in the state. The ASCFR shows an increasing pattern with age. It is as low as 0.14 in the age group 10-year and reaches 7% by age 60 and 11% in the age group 80 years and above.

**Fig. 2:**
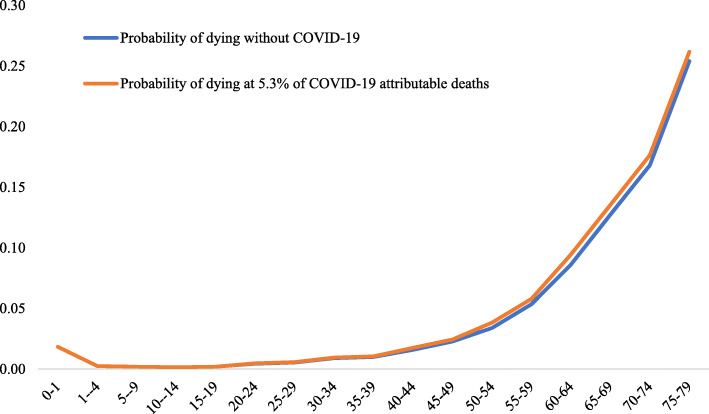
Life table probability of death without and with 5.3% COVID-19 attributable deaths in Maharashtra, India 2020

Figure 2 compares the life table probability of deaths with and without the COVID-19 infection in Maharashtra. Age-specific probability of deaths was estimated by assuming that the age pattern of mortality without COVID-19 would have remained the same as during the period 2014–2018. The gap between the two curves shows the difference in probability of death with and without the COVID-19 pandemic. It is observed that the infection has affected the age pattern of mortality. This pattern suggests that after the age of 44, the probability of dying with COVID-19 is increases with age compared to the probability of dying without COVID-19. The probability of death till the age of 44 is similar both pre- and during the COVID-19 pandemic. The probability of dying with COVID-19, compared to without COVID-19, is higher among those aged 45–75 with COVOD-19 compare to without COVID-19.

**Fig 3:**
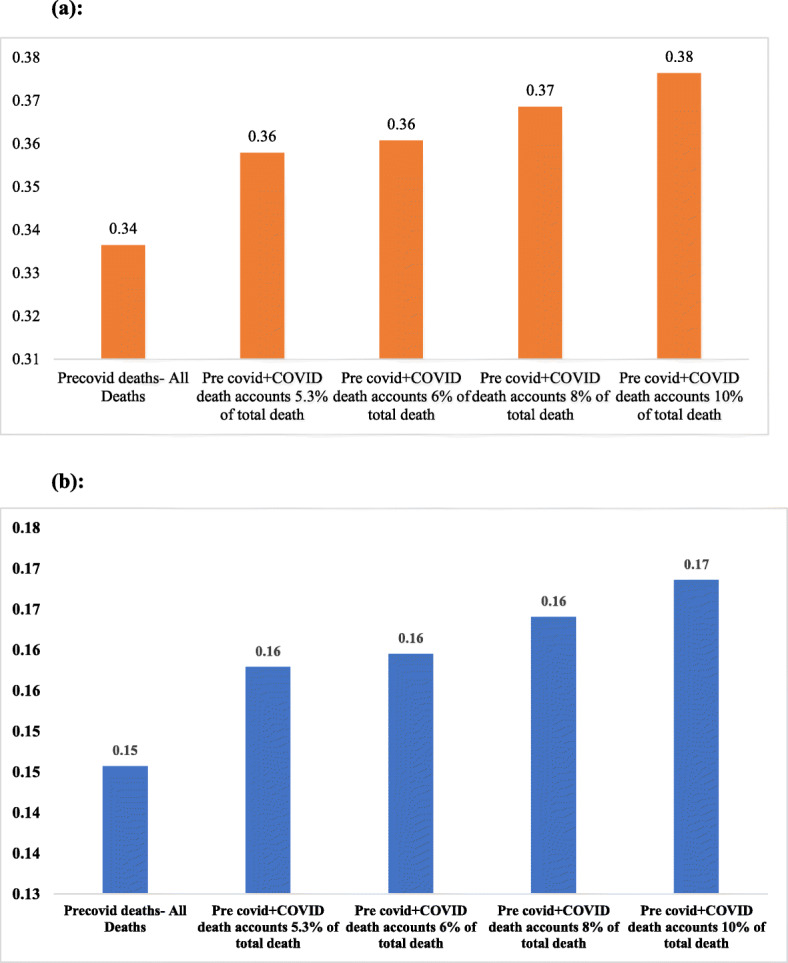
**a** Premature mortality in the age group 0–70 pre- and during COVID-19 pandemic in Maharashtra, India 2020. **b** Premature mortality in the working-age group (20–64) pre- and during COVID-19 pandemic in Maharashtra, India 2020

Figure 3a and b depict the premature mortality by age 70 (${{}_{70}{}q}_0\Big)$ and in the prime working-age group of 20–64 (${{}_{44}{}q}_{20}\Big)$ in pre- and during the COVID-19 pandemic. The premature mortality pre-COVID-19 was 0.34 but increased to 0.36 with COVID-19. Given the current mortality pattern, if the share of deaths attributable to the infection reaches 8 and 10%, the premature mortality (${{}_{70}{}q}_0$) would increase to 0.37 and 0.38, respectively. In the working-age group (${{}_{44}{}q}_{20}\Big)$, the probability of death due to the current rate of infection has increased to 0.16 from 0.15 in the pre-COVID-19 period. Under the assumed 10% COVID-19 attributable death share scenario, the probability of death in the working-age group would increase to 0.17.

Table 2 presents the estimates of life expectancy by age group without COVID-19 and with varying degrees of COVID-19 attributable deaths in Maharashtra. Life expectancy in each age group with and without the COVID-19 infection exhibits the changing age-specific survival pattern. It can be observed that life expectancy has reduced in each scenario of the COVID-19 infection. In the pre-COVID-19 period, life expectancy at birth (age 0) was 73.2 years, which with the current infection, has reduced to 72.4 years. Therefore, life expectancy has reduced by 0.8 years in the state, A disproportionate reduction of life expectancy was observed across age. With an increase in the share of COVID-19 attributable deaths to 10%, life expectancy would reduce to 71.8 years in the state.

**Table 2:** Life expectancy under various scenarios of COVID-19 attributable mortality in Maharashtra, India 2020

Age group	Life expectancy without COVID-19	Life expectancy with COVID-19 deaths of 5.3%	Life expectancy with COVID-19 deaths of 6%	Life expectancy with COVID-19 deaths of 8%	Life expectancy with COVID-19 deaths of 10%	Reduction in life expectancy (pre COVID-19 to 5.3% COVID-19 attributable deaths)
0–1	73.2	72.4	72.3	72.1	71.8	0.8
1–4	73.5	72.8	72.7	72.4	72.1	0.8
5–9	69.7	69.0	68.9	68.6	68.3	0.8
10–14	64.9	64.1	64.0	63.7	63.4	0.8
15–19	59.9	59.2	59.1	58.8	58.5	0.8
20–24	55.1	54.3	54.2	53.9	53.6	0.8
25–29	50.3	49.5	49.4	49.2	48.9	0.8
30–34	45.6	44.8	44.7	44.4	44.2	0.8
35–39	41.0	40.2	40.1	39.9	39.6	0.7
40–44	36.3	35.6	35.5	35.3	35.0	0.7
45–49	31.9	31.2	31.1	30.9	30.6	0.7
50–54	27.6	26.9	26.9	26.6	26.4	0.6
55–59	23.4	22.9	22.8	22.6	22.4	0.5
60–64	19.6	19.2	19.1	18.9	18.8	0.5
65–69	16.2	15.9	15.9	15.7	15.6	0.3
70–74	13.3	13.0	13.0	12.9	12.8	0.3
75–79	10.4	10.2	10.2	10.2	10.1	0.2
80+	8.1	8.0	8.0	7.9	7.9	0.1

**Fig. 4:**
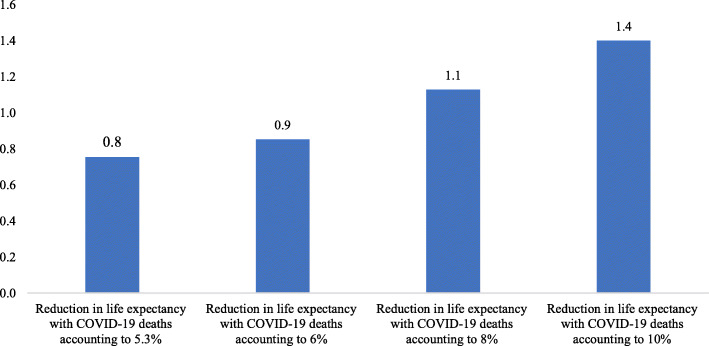
Reduction in life expectancy at birth (in years) due to COVID-19 attributable deaths in Maharashtra, India 2020

Figure 4 shows the reduction in life expectancy at birth in various scenarios of COVID-19 attributable deaths in Maharashtra. Estimates suggest that the ongoing COVID-19 pandemic has significantly affected the life expectancy in the state. Life expectancy has already shrunk by 0.8 years due to the current level of COVID-19 attributable deaths. in the scenario that COVID-19 attributable deaths would amount to 6, 8, and 10% of total deaths in the state, the life expectancy at birth would reduce by 0.9, 1.1, and 1.4 years respectively.

Table 3 presents the estimates of years of potential life lost (YPLL) without COVID-19 and with various level of deaths attributable to COVID-19 by age group in Maharashtra. YPLL was estimated at 17.4 million in the absence of infection. COVID-19 added more than 1.06 million YPLL loss in the state. It can be observed that the age composition of YPLL with infection is substantially different without infection. COVID-19 has significantly affected the working adults aged 45–65 years with their percentage share of YPLL increased to 50% compare to 33% without infection. In contrast, the percentage share of YPLL among infants (0–1 years of age) has decreased to 0.03% with COVID-19 compare to 6.5% without it. In the scenario that COVID-19 attributable deaths increase to 6, 8 and 10%, YPLL would increase by 1.2 million, 1.5 million and 1.9 million respectively.

**Table 3:** Estimates of years of potential life lost (YPLL) in various scenarios of COVID-19 attributable deaths in Maharashtra, India 2020

Age group	Without COVID-19		At 5.3% deaths attributable to COVID-19		At 6% deaths attributable to COVID-19		At 8% deaths attributable to COVID-19		At 10% deaths attributable to COVID-19	
	YPLL	Share of YPLL	YPLL	Share of YPLL	YPLL	Share of YPLL	YPLL	Share of YPLL	YPLL	Share of YPLL
0–1	1,141,269	6.56	335	0.03	379	0.03	503	0.03	627	0.03
1–4	334,770	1.92	3028	0.28	3427	0.29	4552	0.29	5669	0.29
5–9	245,442	1.41	3328	0.31	3767	0.31	5002	0.31	6227	0.32
10–14	185,706	1.07	6445	0.61	7294	0.61	9683	0.61	12,051	0.61
15–19	243,737	1.40	6339	0.60	7173	0.60	9518	0.60	11,842	0.60
20–24	546,719	3.14	25,006	2.35	28,292	2.35	37,528	2.36	46,671	2.37
25–29	617,269	3.55	23,073	2.17	26,100	2.17	34,605	2.18	43,017	2.18
30–34	894,629	5.14	60,284	5.67	68,180	5.67	90,350	5.68	112,252	5.69
35–39	822,372	4.73	49,804	4.68	56,317	4.69	74,590	4.69	92,622	4.70
40–44	1,037,829	5.96	110,039	10.35	124,399	10.35	164,647	10.35	204,310	10.36
45–49	1,163,562	6.69	85,711	8.06	96,879	8.06	128,160	8.06	158,954	8.06
50–54	1,320,430	7.59	172,733	16.25	195,187	16.24	258,010	16.23	319,757	16.21
55–59	1,470,710	8.45	120,684	11.35	136,372	11.35	180,265	11.34	223,405	11.33
60–64	1,576,967	9.06	155,767	14.65	175,994	14.64	232,553	14.62	288,100	14.61
65–69	1,533,804	8.81	100,527	9.45	113,620	9.45	150,284	9.45	186,362	9.45
70–74	13,263,76	7.62	71,380	6.71	80,700	6.71	106,823	6.72	132,571	6.72
75–79	1,203,351	6.92	40,928	3.85	46,286	3.85	61,321	3.86	76,165	3.86
80+	1,735,454	9.97	27,831	2.62	31,478	2.62	41,718	2.62	51,836	2.63
Total	17,400,395	100.0	1,063,242	100.0	1,201,843	100.0	1,590,112	100.00	1,972,438	100.00
0–44	6,069,743	34.88	2876,80	27.06	325,328	27.07	430,979	27.10	535,288	27.14
45–64	5,531,668	31.79	534,895	50.31	604,432	50.29	798,988	50.25	990,216	50.20
65+	5,798,985	33.33	240,667	22.64	272,083	22.64	360,145	22.65	446,934	22.66
Rate of YPLL	140.37		8.58		9.70		12.83		15.91	

Table 4 shows the estimated age-specific DALY in various scenarios of COVID-19 attributable mortality in Maharashtra. With the current share of deaths attributable to the infection, we estimated DALYs for all ages to be 6.1 per thousand population. DALY would change in each scenario of COVID-19 attributable deaths. If COVID-19 accounts for a 6% death share, DALYs would increase to 7 per thousand population. With an increase in the share of deaths attributable to COViD-19 to 8 and 10%, DALYs would increase to 9.2 and 11.5 per thousand population respectively. The age pattern of DALY suggests that the age group 60–64 makes the highest contribution to the overall DALY.

**Table 4:** Age pattern of years of life lost (YLL), years lived with disability (YLD) and disability adjusted life years (DALY) (per 1000 population) in alternative scenarios of COVID-19 infection in Maharashtra, 2020

Age Group	YLL per 1000 population				YLD per 1000 population				DALY per 1000 population			
	5.3% attributable deaths	6% attributable deaths	8% attributable deaths	10% attributable deaths	5.3% attributable deaths	6% attributable deaths	8% attributable deaths	10% attributable deaths	5.3% attributable deaths	6% attributable deaths	8% attributable deaths	10% attributable deaths
0–1	0.16	0.18	0.25	0.31	0.01	0.02	0.02	0.03	0.18	0.20	0.27	0.33
1–4	0.16	0.18	0.25	0.31	0.01	0.02	0.02	0.03	0.18	0.20	0.27	0.33
5–9	0.16	0.18	0.24	0.30	0.01	0.02	0.02	0.03	0.17	0.20	0.26	0.33
10–14	0.30	0.34	0.45	0.57	0.02	0.03	0.04	0.04	0.32	0.37	0.49	0.61
15–19	0.29	0.33	0.44	0.55	0.02	0.03	0.04	0.04	0.32	0.36	0.48	0.60
20–24	1.12	1.27	1.70	2.12	0.05	0.06	0.08	0.10	1.18	1.33	1.78	2.22
25–29	1.08	1.23	1.64	2.05	0.05	0.06	0.08	0.10	1.14	1.29	1.72	2.15
30–34	3.06	3.47	4.63	5.79	0.07	0.08	0.11	0.13	3.13	3.55	4.74	5.92
35–39	2.91	3.30	4.39	5.49	0.07	0.08	0.11	0.13	2.98	3.38	4.50	5.63
40–44	7.66	8.68	11.57	14.47	0.07	0.08	0.11	0.14	7.73	8.77	11.69	14.61
45–49	7.10	8.05	10.74	13.42	0.07	0.08	0.11	0.14	7.18	8.14	10.85	13.56
50–54	17.33	19.64	26.19	32.73	0.09	0.10	0.14	0.17	17.42	19.74	26.32	32.90
55–59	15.55	17.63	23.51	29.38	0.09	0.10	0.14	0.17	15.64	17.73	23.64	29.55
60–64	27.02	30.63	40.84	51.05	0.10	0.11	0.15	0.18	27.12	30.74	40.99	51.23
65–69	23.42	26.55	35.40	44.25	0.10	0.11	0.15	0.18	23.52	26.66	35.54	44.43
70–74	22.02	24.96	33.28	41.60	0.08	0.09	0.12	0.15	22.10	25.05	33.40	41.75
75–79	18.03	20.43	27.24	34.06	0.08	0.09	0.12	0.15	18.11	20.52	27.36	34.20
80+	14.46	16.39	21.86	27.32	0.07	0.08	0.10	0.13	14.53	16.47	21.96	27.45
Total	6.07	6.88	9.17	11.46	0.06	0.06	0.09	0.11	6.12	6.94	9.26	11.57

## Discussion and conclusion

In a short period of 1 year, the COVID-19 pandemic has emerged as the largest-ever health crisis globally, nationally, and locally. Despite several measures to contain the spread of the virus, the infection has intensified the disease burden, increased premature mortality, increased short- and long-term morbidity, raised health care costs, reduced income, increased unemployment, and above all, generated a psychological scare worldwide. The second and the third waves of COVID-19 infection are underway in many of the worst-affected countries. The geographical spread of the COVID-19 infection and the associated mortality has largely been uneven. Its impact varies across and within the countries. India is the second-worst affected country with respect to the number of COVID-19 infections, where the disease remains a constant threat due to the large population, densely populated cities, slums, compromised hygiene practices, inadequate health care facilities, and the lack of other development indicators. The COVID-19 infection rate and fatalities are quite uneven across India, with Maharashtra being the worst affected state, accounting for one-third of the total COVID-19 attributable deaths. In this context, this is the first-ever study to examine the impact of COVID-19 on life expectancy, premature mortality, and disability-adjusted life years in Maharashtra, India. The following are the salient findings.

First, the COVID-19 infection accounted for 5.3% of all causes of death in the state until 20th December 2020. The crude death rate has increased from 7 per thousand in the pre-COVID-19 period to 7.4 per thousand population during COVID-19, which is not a minor shift. A similar finding has been reported in a study from USA [37]. Second, the COVID-19 epidemic has significantly raised the likelihood of dying in the age group 45–70, comprising a large majority of the working adult population. At the current infection rate, the share of premature mortality among working adults (20–64) is 16, and 36% before age 70. In a hypothetical scenario where the level of COVID-19 attributable deaths increase to 10%, the probability of dying prematurely in the age group (0–70) and in the working-age group of 20–64 would rise to 38 and 18% respectively. Similar findings have been reported by various studies across the globe [38, 39]. These observations on the mortality front conveys not only the survival adversity brought about by COVID-19, but also composition of the adversity, which is rather alarming and has long-term implications. Apart from the reversal in the trend of the crude death rate (CDR), the increase in the premature mortality among working adults has resulted in a loss of productivity and put a share of households in distress with the loss of the bread earner. This may lead to a range of adversities like discontinuation of education by children and debt burden among distressed households with gendered derivatives, wherein dependent girls, children, and women will become worse off. Premature mortality, unless otherwise sufficiently protected with insurance and economic protection can have a devastating impact on individual households, which may not be apparent in the macro scene. Third, with the current share of COVID-19 induced mortality, life expectancy has already shrunk by 0.8 years in the state. If the virus continues to spread and mortality reaches 10%, loss in life expectancy is likely to be 1.4 years. Our findings are consistent with literature [4, 8, 18]. A high reduction of life expectancy (2.94 years) has been observed in USA [40]. Fourth, The COVID-19 attributable deaths have caused about 1.06 million YPLL in the state. The majority of the loss in YPLL has been among the working adults aged 45–64 years. This disproportionately high share of person-years life lost in the ages 45–64 has undoubtedly increase the dependency burden at the household level, which calls for micro-monitoring of such households and adoption of appropriate protective measures for the dependents of the adult victims. Lastly, the COVID-19 induced mortality has substantial implications for DALY as well. At the current share of COVID-19 induced mortality, the loss of DALY was estimated at 6.1 per thousand population. With an increase in the deaths share to 8 and 10% of mortality, DALY loss is estimated to increase to 9.2 and 11.5 per thousand population, respectively.

The new strains of COVID-19 at various places across the globe have alarmed the world about the higher transmission probability and higher associated mortality than the existing strains of virus [41]. Therefore, advanced preparedness needs to be in place to tackle the rapid spread of the infection until a substantial share of the population is vaccinated. Since the beginning of the pandemic, the national and states governments in India have made several efforts to contain the spread of the virus with measures like imposing a complete lockdown, promoting hygiene and hand wash practices, executing a phase-wise unlocking, promoting social distancing norms and mask wearing, identifying hot spots, and others. Though the infection rate had slowed down in the country, COVID-19 infection is surging again, whereas Maharashtra continues to be the worst affected state in the country. In recent months, Maharashtra has accounted for about half of the new COVID-19 infections in the country. The likely reasons behind this is the densely populated cities, the presence of a large number of slums, the large inflow and outflow of migrants, the engagement of a substantial labour force in the unorganized sector, along with a number of demographic challenges. The potential for rapid transmission and the implied fatalities may well reverse the survival scene in the state as indicated by this study [12].

It is heartening to note that India has been successful in developing two of the vaccines, namely ‘Covaxin’ and ‘Covishield’, and started the vaccination exercise on 16th January 2021. As of 5th March 2021, more than 15 million people have been vaccinated in the country of whom 1.4 million are in the state of Maharashtra [22]. The current vaccination program is limited to health care workers, senior citizens and persons with comorbidities. Giving the fact that half of the newly infected cases are from Maharashtra, it is suggested that the state be given priority in vaccination. The vaccines are being supplied by the central government while the vaccinated are being carried out by the state governments. The state of Maharashtra and the cities in the state should be accorded a high priority in the vaccination program. Also the IEC (Information Education and Communication) on the efficacy of the vaccination should be intensified to reduce the vaccine hesitancy and to eliminate the mistrust among some sections of the population.

Given that the risk of The COVID-19 infection is age conditioned, with the vulnerability being greater among those aged 40 and above, and that the virulence of the disease intensifies with multimorbid conditions and a compromised immunity, the rate of infection has been quite high in the later ages, with an increased risk of fatality as well. In view of the need for out-of-home activities for livelihood and the prevalence of inappropriate working conditions that hardly allow for COVID protocols of SMS (Sanitary Practice, Masking and Safe Social Distance) to be followed, there needs to be a greater focus on this vulnerable population to attain a balance between work and life.

In conclusion, this exercise makes a precise assessment of the survival scenario keeping in mind the continuation of the COVID-19 pandemic. The study emphasize the need for robust protection measures to mitigate the consequences of the disease on victim households and for prioritizing the vaccination program in the state of Maharashtra. The very specific vulnerability to this infection calls for suitable action on a variety of fronts like work environments out of home, means of communication in keeping with the COVID-19 protocols, and adequate sanitary and hygiene amenities in the living environment to restrict the spread of the infection and to bring it under control before prior to the vaccination drive goes into in full swing.

## Supplementary Information

**Additional file 1: Appendix 1.** Age-specific COVID-19 confirmed cases, deaths and estimated case fatality ratio (CFR) in Maharashtra, 20th December, 2020. **Appendix 2.** Estimated deaths without COVID-19 and with varying level of COVID-19 attributable deaths in Maharashtra, India, 20th December, 2020.

## Data Availability

Multiple data sources were used in the current study. The data is available in the public domain and is accessible online at: https://api.covid19india.org/, https://nhm.gov.in/New_Updates_2018/Report_Population_Projection_2019.pdf, https://censusindia.gov.in/vital_statistics/Appendix_SRS_Based_Life_Table.html, https://timesofindia.indiatimes.com/city/mumbai/one-in-3-covid-deaths-across-maharashtra-was-of-person-in-61-70-age-group/articleshow/79599564.cms, https://timesofindia.indiatimes.com/city/mumbai/maharashtra-mumbai-see-slight-drop-in-cases-marginal-rise-in-deaths/articleshow/79831787.cms
