# Supplementary material for: Impact of COVID-19 infection on life expectancy, premature mortality, and DALY in Maharashtra, India
Source: BMC Infect Dis. 2021 Apr 12;21:343. doi: 10.1186/s12879-021-06026-6 (PMC8040360; doi:10.1186/s12879-021-06026-6)
Supplement: Supplementary file 1 — Additional file 1: Appendix 1. Age-specific COVID-19 confirmed cases, deaths and estimated case fatality ratio (CFR) in Maharashtra, 20th December, 2020. Appendix 2. Estimated deaths without COVID-19 and with varying level of COVID-19 attributable deaths in Maharashtra, India, 20th December, 2020. [file 12879_2021_6026_MOESM1_ESM.docx]

**Impact of COVID-19 infection on life expectancy, premature mortality, and DALY in Maharashtra, India**

**Guru Vasishtha^1^, Sanjay K. Mohanty^2^, Udaya S. Mishra^3^, Manisha Dubey^4^, and Umakanta Sahoo^5^**

**Corresponding Author**

Guru Vasishtha

Research Scholar,

International Institute for Population Sciences

Govandi Station Road, Deonar, Mumbai-400088,

Maharashtra, India.

**Orchid id: 0000-0001-5477-1845**

**Email:** [**guruvasishth15@gmail.com**](mailto:guruvasishth15@gmail.com)

**^1^ Research Scholar, International Institute for Population Sciences, Mumbai, India**. Email: [guruvasishth15@gmail.com](mailto:guruvasishth15@gmail.com)

^2^ Professor, Department of Fertility Studies, International Institute for Population Sciences, Mumbai, India. Email: [sanjayiips@yahoo.co.in](mailto:sanjayiips@yahoo.co.in)

^3^ Professor, Centre for Development Studies, Prashant Nagar, Medical College P.O, Ullor Thiruvananthapuram, Kerala, India. Email: [udayamishra64@gmail.com](mailto:udayamishra64@gmail.com)

^4^ Senior Research Associate, Centre for Chronic Disease Control, New Delhi, India. Email: [manikvdlw@gmail.com](mailto:manikvdlw@gmail.com)

5 Research Scholar, International Institute for Population Sciences, Mumbai, India. Email: sahooumakanta0@gmail.com

**Appendix 1:** Age-specific COVID-19 confirmed cases, deaths and estimated case fatality ratio (CFR) in Maharashtra, 20^th^ December, 2020.

| **Age Group** | **Confirmed cases** | **Covid19 death** | **ASCFR(%) (estimated)** |
| --- | --- | --- | --- |
| 0-1 | 3,252 | 5 | 0.14 |
| 1-4 | 29,267 | 42 | 0.14 |
| 5-9 | 33,953 | 48 | 0.14 |
| 10--14 | 61,540 | 101 | 0.16 |
| 15-19 | 65,536 | 107 | 0.16 |
| 20-24 | 157,405 | 461 | 0.29 |
| 25-29 | 159,173 | 466 | 0.29 |
| 30-34 | 209,294 | 1,345 | 0.64 |
| 35-39 | 192,646 | 1,238 | 0.64 |
| 40-44 | 180,687 | 3,089 | 1.71 |
| 45-49 | 160,611 | 2,746 | 1.71 |
| 50-54 | 168,859 | 6,413 | 3.80 |
| 55-59 | 138,706 | 5,268 | 3.80 |
| 60-64 | 117,433 | 8,128 | 6.92 |
| 65-69 | 91,336 | 6,322 | 6.92 |
| 70-74 | 57,935 | 5,492 | 9.48 |
| 75-79 | 42,135 | 3,994 | 9.48 |
| 80+ | 31,887 | 3,482 | 10.92 |
| **Total** | **1,901,654** | **48,746** | **2.56** |

**Source:** Times of India dated 7^th^ December 2020 and Times of India dated 21^st^ December 2020.

**Appendix 2:** Estimated deaths without COVID-19 and with varying level of COVID-19 attributable deaths in Maharashtra, India, 20^th^ December, 2020.

| **Age group** | **Population (in 000)** | **ASDR (SRS, 2014-18) without COVID-19** | **Distribution of estimated number of deaths without COVID-19** | **% distribution of COVID-19 death** | **Distribution of actual deaths with COVID deaths** | | | **Estimated total deaths** | | | **ASDR with COVID-19** | | | **Probability of Dying (_n_q_x_)** | | | |
| --- | --- | --- | --- | --- | --- | --- | --- | --- | --- | --- | --- | --- | --- | --- | --- | --- | --- |
|  |  |  |  |  |  |  |  |  |  |  |  |  |  |  |  |  |  |
|  |  |  |  |  |  |  |  |  |  |  |  |  |  |  |  |  |  |
|  |  |  |  |  | **share at 5.3%** | **share at 6%** | **share at 8%** | **at 5.3 % COVID attributable deaths** | **at 6% COVID attributable deaths** | **at 8% COVID attributable deaths** | **share at 5.3 %** | **share at 6 %** | **share at 8 %** | **without COVID-19** | **at 5.3% of Covid-19 attributable death** | **at 6% of Covid-19 attributable death** | **at 8% of Covid-19 attributable death** |
|  | **(col 1)** | **(col 2)** | **(col 3)** | **(col 4)** | **(Col 5)** | **(Col 6)** | **(Col 7)** | **(Col 8)** | **(Col 9)** | **(Col 10)** | **(Col 11)** | **(Col 12)** | **(Col 13)** | **(col 14)** | **(Col 15)** | **(Col 16)** | **(Col 17)** |
| 0-1 | 843 | 18.5 | 15,594 | 0.0001 | 5 | 5 | 7 | 15,599 | 15,600 | 15,601 | 18.5 | 18.5 | 18.5 | 0.0183 | 0.0183 | 0.0183 | 0.0183 |
| 1--4 | 75,86 | 0.6 | 4,552 | 0.0009 | 42 | 47 | 63 | 4,593 | 4,599 | 4,615 | 0.6 | 0.6 | 0.6 | 0.0024 | 0.0024 | 0.0024 | 0.0024 |
| 5--9 | 8,801 | 0.4 | 3,520 | 0.0010 | 48 | 55 | 73 | 3,569 | 3,575 | 3,593 | 0.4 | 0.4 | 0.4 | 0.0020 | 0.0020 | 0.0020 | 0.0020 |
| 10--14 | 9,545 | 0.3 | 2,863 | 0.0021 | 101 | 114 | 152 | 2,964 | 2,977 | 3,015 | 0.3 | 0.3 | 0.3 | 0.0015 | 0.0016 | 0.0016 | 0.0016 |
| 15-19 | 10,165 | 0.4 | 4,066 | 0.0022 | 107 | 121 | 162 | 4,173 | 4,187 | 4,228 | 0.4 | 0.4 | 0.4 | 0.0020 | 0.0021 | 0.0021 | 0.0021 |
| 20-24 | 11,033 | 0.9 | 9,929 | 0.0094 | 461 | 522 | 696 | 10,390 | 10,451 | 10,625 | 0.9 | 0.9 | 1.0 | 0.0045 | 0.0047 | 0.0047 | 0.0048 |
| 25-29 | 11,156 | 1.1 | 12,272 | 0.0096 | 466 | 528 | 704 | 12,738 | 12,800 | 12,976 | 1.1 | 1.1 | 1.2 | 0.0055 | 0.0057 | 0.0057 | 0.0058 |
| 30-34 | 10,909 | 1.8 | 19,635 | 0.0276 | 1345 | 1525 | 2033 | 20,981 | 21,160 | 21,669 | 1.9 | 1.9 | 2.0 | 0.0090 | 0.0096 | 0.0097 | 0.0099 |
| 35-39 | 10,041 | 2 | 20,082 | 0.0254 | 1238 | 1404 | 1871 | 21,320 | 21,485 | 21,953 | 2.1 | 2.1 | 2.2 | 0.0100 | 0.0106 | 0.0106 | 0.0109 |
| 40-44 | 8,925 | 3.2 | 28,561 | 0.0634 | 3089 | 3501 | 4668 | 31,650 | 32,062 | 33,229 | 3.5 | 3.6 | 3.7 | 0.0159 | 0.0176 | 0.0178 | 0.0184 |
| 45-49 | 7,934 | 4.6 | 36,494 | 0.0563 | 2746 | 3112 | 4150 | 39,240 | 39,606 | 40,644 | 4.9 | 5.0 | 5.1 | 0.0227 | 0.0244 | 0.0247 | 0.0253 |
| 50-54 | 6,942 | 6.9 | 47,899 | 0.1316 | 6413 | 7269 | 9692 | 54,312 | 55,168 | 57,591 | 7.8 | 7.9 | 8.3 | 0.0339 | 0.0384 | 0.0390 | 0.0406 |
| 55-59 | 5,702 | 11 | 62,724 | 0.1081 | 5268 | 5971 | 7961 | 67,992 | 68,695 | 70,686 | 11.9 | 12.0 | 12.4 | 0.0535 | 0.0579 | 0.0585 | 0.0601 |
| 60-64 | 4,463 | 18 | 80,327 | 0.1668 | 8128 | 9213 | 12284 | 88,455 | 89,540 | 92,611 | 19.8 | 20.1 | 20.8 | 0.0861 | 0.0944 | 0.0955 | 0.0986 |
| 65-69 | 3,471 | 27.2 | 94,409 | 0.1297 | 6322 | 7166 | 9554 | 100,731 | 101,575 | 103,963 | 29.0 | 29.3 | 30.0 | 0.1273 | 0.1353 | 0.1363 | 0.1393 |
| 70-74 | 2,727 | 36.7 | 100,086 | 0.1127 | 5492 | 6225 | 8300 | 105,578 | 106,311 | 108,386 | 38.7 | 39.0 | 39.7 | 0.1681 | 0.1765 | 0.1776 | 0.1808 |
| 75-79 | 1,983 | 58.2 | 115,432 | 0.0819 | 3994 | 4527 | 6036 | 119,427 | 119,960 | 121,469 | 60.2 | 60.5 | 61.2 | 0.2540 | 0.2617 | 0.2627 | 0.2656 |
| 80+ | 1,735 | 123.1 | 213,634 | 0.0714 | 3482 | 3946 | 5262 | 217,116 | 217,581 | 218,896 | 125.1 | 125.4 | 126.1 | 1.0000 | 1.0000 | 1.0000 | 1.0000 |
| **Total** | 123,961 |  | 872,081 | 1.0000 | 48746 | 55252 | 73669 | 920,827 | 927,332 | 945,750 |  |  |  |  |  |  |  |

Note: Col 1: Report of the Expert Committee on Population Projections 2019, Col 2: SRS, 2014-17, Col 3=col1*col2, col 4: covid19india.org
